# A Case Study of Abdominal Aortic Aneurysm Detection and Critical Vascular Surgery

**DOI:** 10.7759/cureus.58894

**Published:** 2024-04-24

**Authors:** Mamta Kamboj, Arghadip Das, Hadeeqa Idris, Ajay Singh, Sachin M Chaudhary, Mohitha C Mallipeddi

**Affiliations:** 1 Internal Medicine, Dayanand Medical College and Hospital, Ludhiana, IND; 2 Internal Medicine, Nil Ratan Sircar Medical College and Hospital, Kolkata, IND; 3 Internal Medicine, Shifa International Hospital Islamabad, Islamabad, PAK; 4 Internal Medicine, Shri Ram Murti Smarak Institute of Medical Sciences, Bareilly, IND; 5 Internal Medicine, Gujarat Cancer Society (GCS) Medical College, Hospital and Research Centre, Ahmedabad, IND; 6 Internal Medicine, Sri Venkateswara Institute of Medical Sciences (SVIMS), Tirupati, IND

**Keywords:** vascular surgery, early detection, risk factors, screening, endovascular aneurysm repair, abdominal aortic aneurysm

## Abstract

Abdominal aortic aneurysm, characterized by a persistent dilation exceeding 3 cm or 50% of the aortic diameter, poses a substantial risk, particularly in males over 65. Despite its potentially asymptomatic nature, early detection is imperative due to the elevated mortality rates, reaching 90% following rupture. The presented case involves a 60-year-old male with progressively worsening abdominal pain, a history of cardiovascular disease, hypertension, and smoking. Initial examinations were inconclusive, requiring advanced imaging that revealed a large aneurysmal dilation. Therapeutic measures included endovascular aneurysm repair (EVAR), highlighting the significance of timely intervention. Despite elective surgery risks, mortality rates decrease significantly when the aneurysm diameter surpasses 43 mm. This report stresses the need for primary care physicians to conduct thorough screenings, recognize risk factors, and facilitate prompt referrals for advanced imaging. The case's pivotal lesson lies in the comprehensive management of abdominal aortic aneurysm, showcasing the potential for life-saving interventions and the critical role of early detection in mitigating the severe consequences associated with its rupture.

## Introduction

A persistent dilatation of the abdominal aorta with a diameter greater than 3 cm or greater than 50% of the aortic diameter at the diaphragmatic level is indicative of an abdominal aortic aneurysm (AAA) [1]. Usually located inferiorly to the renal arteries in 88-89% of cases, an AAA results from gradual vascular wall deterioration that causes dilating and weakening if left untreated [2]. Atherosclerosis, smoking, male gender, advancing age, Caucasian ethnicity, having a family history of AAA, hypertension, high cholesterol, and an existing history of aortic dissection are the main risk factors [3].

AAA is the ninth most common cause of death for men over 65 [4]. The size of the aneurysm determines the rupture risk, and rupture results in a mortality rate higher than 80% [5]. The most common clinical indication of AAA is a pulsatile, painless abdominal mass, but it can also stay asymptomatic despite its potential severity [6]. Because AAA is usually quiet in its early stages and rupture can have serious implications, early detection and care are critical. We describe a fissured aortic aneurysm case to highlight the critical importance of screening, referral for vascular surgery, and life-saving measures.

## Case presentation

A 60-year-old man presented to the emergency department with a complaint of progressively increasing abdominal pain and distension over the past four months. His medical history was notable for known cardiovascular disease, hypertension, and a history of smoking. Upon presentation, the patient's vital signs were stable, with a blood pressure of 130/90, a pulse rate of 58/min, a respiratory rate of 17/min, an oxygen saturation of 98% on room air, and a temperature of 36.5°C. The patient's body mass index measured 26.29 kg/m^2^. Physical examination findings revealed clear lungs, a regular heart rate and rhythm, and an obese abdomen characterized by softness and mild distension. Palpation did not detect any AAA or hepatosplenomegaly. However, the patient experienced significant discomfort during light palpation of the abdomen.

The laboratory findings were generally within normal limits. Notably, the patient's anticoagulation was below the therapeutic range, with an international normalized ratio of 1.9 (Table 1).

**Table 1:** Laboratory findings WBC: white blood cell

Laboratory parameter	Finding	Normal range
Troponin I level	0.02 ng/mL	0.00-0.09 ng/mL
WBC count	13.9x10^9^/L	4.5-11.0x10^9^/L
Hematocrit	43%	40-54%
Anticoagulation rate	1.9	2.0-3.0

**Figure 1:**
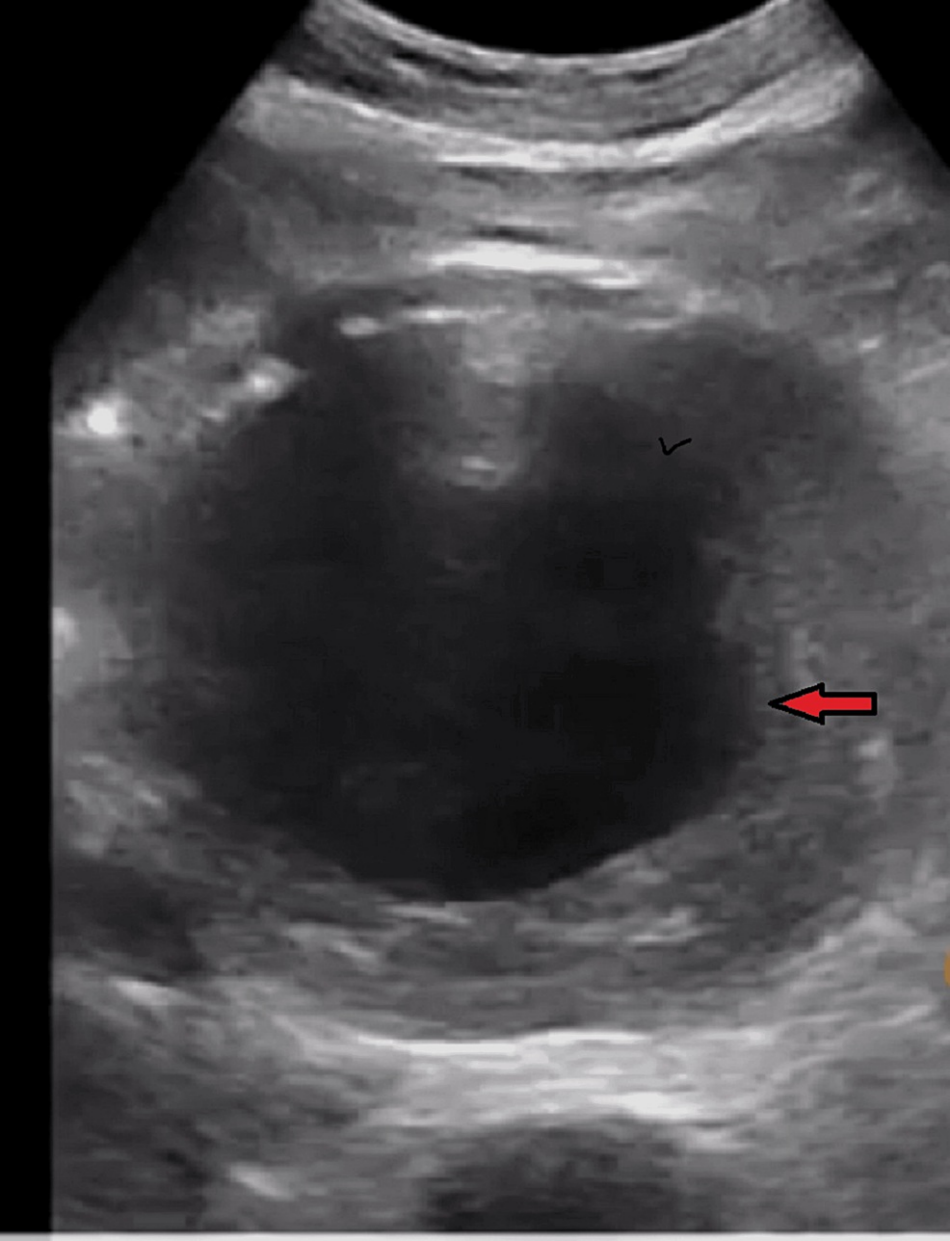
Ultrasound image showing AAA with a lesion measuring 9 cm with circumferential parietal thrombus AAA: abdominal aortic aneurysm

**Figure 2:**
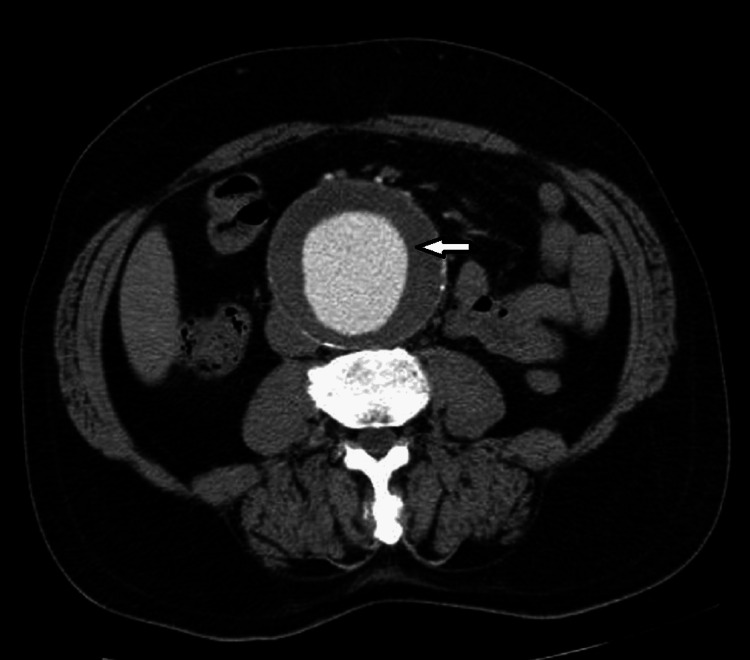
Axial contrast-enhanced computed tomography revealing a significant aneurysmal dilation originating from the posterior of the abdominal aorta below the renal arteries

The electrocardiogram results revealed a normal sinus rhythm without acute ST changes. An abdominal Doppler ultrasound revealed a saccular lesion measuring 9 cm with a circumferential parietal thrombus communicating with the aorta through a 32-mm-wide neck (Figure 1). Subsequent abdominal multi-detector row computed tomography (MDCT) was conducted for a more comprehensive assessment, disclosing a large saccular aneurysmal dilation originating from the posterior of the abdominal aorta below the renal arteries, connected by a 30-mm-wide neck with circumferential parietal thrombosis respecting the arterial lumen (Figure 2). Upon further investigation, it was revealed that the patient's sibling had previously experienced an AAA a few years ago, ultimately succumbing to the consequences of AAA rupture.

**Figure 3:**
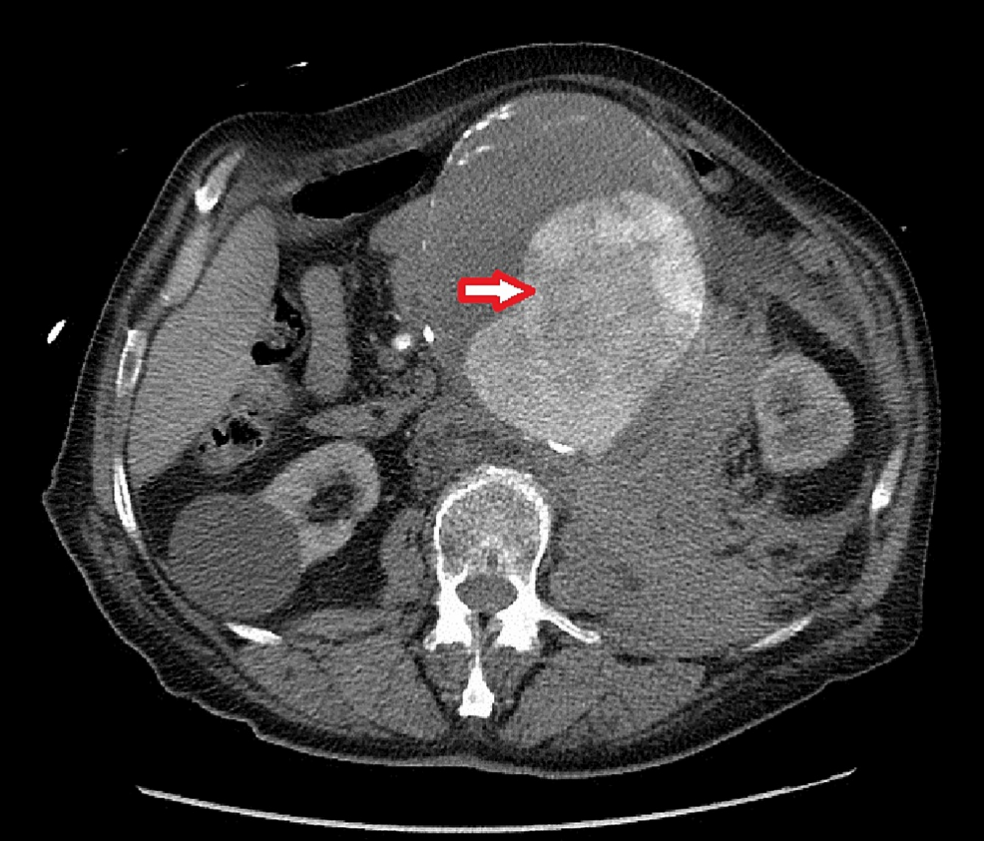
Computed tomography (axial view) showing significant aneurysm in the abdominal aorta with minimal surrounding stranding, suggesting an early or slow rupture. Despite the limited evidence of blood leakage, this condition remains a surgical emergency

The patient returned a few days later with intensified abdominal pain and in a hemodynamically unstable condition. An emergency abdominal-pelvic CT scan revealed an extensive saccular aneurysmal dilation of the infra-renal abdominal aorta, peritoneal effusion, mesenteric infiltration, and parietal hyperdensity, confirming an aneurysmal fissure (Figure 3).

Therapeutic intervention commenced with the establishment of appropriate intravenous access, judicious administration of intravenous fluids, and pain management. Endovascular aneurysm repair (EVAR) was performed, and the patient was prescribed a treatment regimen comprising angiotensin 2 receptor blockers, acetylsalicylic acid, and clopidogrel. The patient's condition and vital signs showed improvement, and a follow-up was advised.

## Discussion

It is estimated that approximately 0.6 million individuals in India were affected by AAA in 2016, with a prevalence of 0.4% [7]. Patients with a ruptured AAA face alarmingly high mortality rates, reaching up to 90%. Conversely, mortality rates for those undergoing elective AAA repair are typically under 10% [8]. Therefore, early AAA diagnosis is crucial to prevent rupture. Common risk factors for AAA include male gender, age over 65, a history of tobacco use, and a familial predisposition [2]. Approximately 4-8% of men and 0.5-2% of women above 60 years old are estimated to have AAA. If left untreated, AAAs tend to progressively enlarge, with larger aneurysms carrying a higher risk of rupture [9]. Moreover, larger aneurysms exhibit a faster rate of expansion compared to smaller ones. AAA repair stands out as a relatively safe and effective method to mitigate the risk of rupture-associated death. Primary care physicians play a crucial role in ordering appropriate AAA screenings and should possess knowledge about when to refer patients to vascular surgeons, optimize pre-surgery comorbidities, understand potential complications, and be aware of post-repair surveillance requirements.

In instances where symptoms manifest, the typical grievances include lower back pain and abdominal discomfort. Recognizing these signs, symptoms, and associated risk factors is crucial due to the frequently inconspicuous nature of the diagnosis, leading to misdiagnosis in 20-30% of cases [10]. In addition to back or abdominal pain, patients can additionally experience pain in the hip, flank, groin, or buttock. Patients may describe this pain as severe or piercing, but it is generally nonspecific if the AAA compresses a nearby structure such as a vertebral endplate [11]. Sudden ischemia, painful cyanotic toes, and palpable pedal pulses are examples of leg symptoms that might come from distal embolization or aortic occlusion caused by thrombosis [12]. More intense pain with an abrupt beginning is caused by rupture or dissection, which occurs when bleeding into the media of the vessel separates its layers [13]. The patient in this case experienced low back pain radiating into the leg but did not exhibit the thrombosis-related signs.

One significant risk factor is smoking, defined as consuming more than 100 cigarettes over the course of a lifetime [14]. Interestingly, the only controllable factor associated with the growth of AAA is quitting smoking. In this specific case, the patient aligns with the risk factors related to age, gender, and a smoking history.

Individuals who are close blood relatives, especially male family members, of a person already diagnosed with an aneurysm face an elevated risk [15]. In our case, the patient's sibling had earlier succumbed to the rupture of AAA. Another correlation observed with AAA is the presence of atherosclerotic disease, encompassing conditions like coronary heart disease and claudication, as seen in the instance of our patient. However, the diagnosis of AAA cannot be conclusively determined by a single risk factor or characteristic, as it is a complex disorder influenced by various genetic and environmental factors.

The clinical examination has restricted effectiveness in identifying AAA. Nonetheless, the researchers emphasize the significance of abdominal palpation and auscultation, particularly when there is a suspicion of non-mechanical or abdominal issues causing low back pain or when patients show resistance to treatment [16]. Another scenario justifying clinical examination is when the patient's clinical history raises concerns about AAA. In this particular case, abdominal palpation was conducted during the initial examination, even though the patient did not exhibit symptoms at that time.

The patient's abdominal circumference and the aneurysm's size have an impact on the detection of AAA [17]. It becomes extremely uncommon for AAAs to be palpable in patients whose girths are more than 100 cm. However, the chance of a clinical diagnosis also rises with the size of the aneurysm. This suggests that among obese patients, a referral for ultrasonography evaluation may be prudent if the medical history discloses enough indications and risk factors. This obese patient had a large AAA, but it was difficult to palpate and detect. Ultrasound is a more reliable screening method for male AAA than abdominal palpation, although it is less accurate for female patients. Research has demonstrated that offering a baseline screening test to males over 65 can cut the population's AAA-related mortality in half [18]. An ultrasonography examination was used to confirm the aneurysm's presence in this patient.

Nonetheless, there are risks associated with elective surgical repair of AAA. Operative death rates range from 1.4% to 5.8%, with a 32.4% complication rate [2]. As a result, aneurysms are usually left untreated surgically until they have a diameter of at least 43 mm; some research even advises against doing surgery until the aneurysm is larger than 50-55 mm [19]. Age alone does not determine operating eligibility; however, patients with concomitant morbidity and those awaiting treatment for AAA have the highest death rates [20].

## Conclusions

Our case underscores the importance of appropriately screening for AAA, referring patients to vascular surgery, and taking into account a high level of suspicion and potentially life-saving measures. There are distinct risk factors and unique symptoms associated with AAA. Despite the relatively low sensitivity of such evaluations, a clinical assessment is necessary when identified indicators of risk are visible. For any male patient 50 years of age or older with low back pain, it should be part of the differential diagnosis. It is advised to promptly refer the patient for advanced imaging if there is any suspicion.
